# Unmet needs in hereditary angioedema: an international survey of physicians

**DOI:** 10.1186/s13023-025-03739-8

**Published:** 2025-07-28

**Authors:** Thomas Buttgereit, Felix Aulenbacher, Adil Adatia, Carolina Vera Ayala, Maryam Ali Al-Nesf, Sabine Altrichter, Mohamed Abuzakouk, Mona Al-Ahmad, Ramzy Mohammed Ali, Alejandro Berardi, Isabelle Boccon-Gibod, Laurence Bouillet, Luisa Brussino, Marko Barešić, Paula J. Busse, Stephen D. Betschel, Herberto Chong-Neto, Oscar Calderón Llosa, Timothy J. Craig, Anthony D. Dorr, Sérgio Duarte Dortas Junior, Daria Fomina, Henriette Farkas, Jie Shen Fok, Anete S. Grumach, Jens Greve, Mar Guilarte, Margarida Gonçalo, Vesna Grivcheva-Panovska, Michihiro Hide, Roman Hakl, Ankur Jindal, Constance H. Katelaris, Shailajah Kamaleswaran, Tamar Kinaciyan, Elena Latysheva, José Ignacio Larco Sousa, Ramón Lleonart Bellfill, Hassan Mobayed, Martin Metz, Iman Nasr, Natasa T. Mitrevska, Stefania Nicola, Claudio Alberto Salvador Parisi, Grzegorz Porebski, Jonny Peter, Mariana Paes Leme Ferriani, Nelson Rosario Filho, Bülent Enis Şekerel, Faradiba Sarquis Serpa, Marcin Stobiecki, Susanne Trainotti, Anna Valerieva, Chamard Wongsa, Jane C. Y. Wong, Esra Yucel, Yinglei Li, Chiara Nenci, Marcus Maurer, Markus Magerl, Philip H. Li

**Affiliations:** 1 Dermatology and Allergy, Institute of Allergology, Charité–Universitätsmedizin Berlin, Freie Universität Berlin and Humboldt-Universität Zu Berlin, Hindenburgdamm 27, 12203 Berlin, Germany; 2 Fraunhofer Institute for Translational Medicine and Pharmacology ITMP, Immunology and Allergology, Berlin, Germany; 3 Division of Pulmonary Medicine, Department of Medicine, University of Alberta, Edmonton, AB Canada; 4 Allergy and Immunology Division, Department of Medicine, Hamad Medical Corporation, Doha, Qatar; 5 Department of Dermatology and Venereology, Kepler University Hospital, Linz, Austria; 6 Center for Medical Research, Johannes Kepler Universität Linz, Linz, Austria; 7 Cleveland Clinic, Cleveland, OH USA; 8 Respiratory Institute, Cleveland Clinic Abu Dhabi, Abu Dhabi, United Arab Emirates; 9 Department of Microbiology, College of Medicine, Kuwait University, Kuwait City, Kuwait; 10 Instituto de Asma, Alergia y Enfermedades Respiratorias, GA²LEN UCARE Urticaria Centers of Reference and Excellence, GA²LEN ACARE Angioedema Centers of Reference and Excellence, Corrientes, Argentina; 11 French National Reference Center for Angioedema (CREAK) and ACARE, Internal Medicine Department, Grenoble Alpes University Hospital (CHUGA), Grenoble, France; 12 Grenoble Alpes University, T-RAIG Unit, CNRS, UMR 5525, TIMC, French National Reference Center for Angioedema (CREAK), Internal Medicine Department, Grenoble Alpes University Hospital, Grenoble, France; 13 Immunology and Allergy Department, AO Ordine Mauriziano, Department of Medical Science, University of Turin, Turin, Italy; 14 Division of Clinical Immunology and Rheumatology, Department of Internal Medicine, School of Medicine, University Hospital Center Zagreb, Zagreb, Croatia; 15 Division of Clinical Immunology, Department of Medicine, Mount Sinai, New York, NY USA; 16 Division of Clinical Immunology and Allergy, Department of Medicine, University of Toronto, Toronto, ON Canada; 17 Department of Pediatrics, Federal University of Paraná, Curitiba, Brazil; 18 ACARE Center Clínica, SANNA El Golf, Lima, Peru; 19 Vinmec International Hospital, Times City, Hanoi Vietnam; 20 Department of Clinical Immunology, Barts Health NHS Trust, London, UK; 21 Serviço de Imunologia, Hospital Universitário Clementino Fraga Filho (HUCFF-UFRJ), Rio de Janeiro, Brazil; 22 Moscow City Clinical and Research Center of Allergology and Immunology, City Clinical Hospital 52, Moscow, Russia; 23 GA²LEN UCARE and ACARE Center, Department of Clinical Immunology and Allergology, I.M. Sechenov First Moscow State Medical University (Sechenov University), Moscow, Russia; 24 Department of Pulmonology, Astana Medical University, Astana, Kazakhstan; 25 Hungarian Angioedema Center of Reference and Excellence, Department of Internal Medicine and Haematology, Semmelweis University, Budapest, Hungary; 26 Department of Respiratory Medicine, Box Hill Hospital, Melbourne, Australia; 27 Monash Lung, Sleep, Allergy/Immunology, Monash Medical Centre, Melbourne, Australia; 28 Eastern Health Clinical School, Monash University, Melbourne, Australia; 29 Clinical Immunology, Faculty of Medicine, Centro Universitario FMABC, Santo Andre, Brazil; 30 Department of Otorhinolaryngology, Head and Neck Surgery, Ulm University Medical Center, Ulm, Germany; 31 Department of Allergy, Angioedema Reference Center (CSUR), Hospital Universitari Vall d’Hebron, Institut de Recerca Vall d’Hebron (VHIR), Barcelona, Spain; 32 Dermatology Department, University Hospital, Coimbra Local Health Unit and Faculty of Medicine, University of Coimbra, Coimbra, Portugal; 33 University Clinic of Dermatology, School of Medicine, University St. Cyril and Methodius, Skopje, North Macedonia; 34 Department of Dermatology, Hiroshima City Hiroshima Citizens Hospital, Hiroshima, Japan; 35 Department of Dermatology, Hiroshima University Hospital, Hiroshima, Japan; 36 Department of Clinical Immunology and Allergology, St. Anne’s University Hospital Brno, Brno, Czech Republic; 37 Faculty of Medicine, Masaryk University, Brno, Czech Republic; 38 Allergy Immunology Unit, Department of Pediatrics, Advanced Pediatrics Centre, Post Graduate Institute of Medical Education and Research, Chandigarh, India; 39 Immunology & Allergy Unit, Department of Medicine, Campbelltown Hospital and Western Sydney University, Sydney, Australia; 40 Dermatology and Allergy Center, Odense Universitetshospital, Svendborg Sygehus, Nyborg Sygehus, Denmark; 41 Department of Dermatology, Medical University of Vienna, Vienna, Austria; 42 Immunopathology Department, NRC Institute of Immunology FMBA Russia, Pirogov Russian National Research Medical University, Moscow, Russia; 43 Allergy Department, Clínica San Felipe, Lima, Peru; 44 Allergology Department, Hospital Universitari de Bellvitge, Institut de Recerca IDIBELL L’Hospitalet de Llobregat, Barcelona, Spain; 45 Adult Immunology and Allergy Unit, Royal Hospital, Muscat, Oman; 46 Department of Dermatology, Re-Medika Hospital, Skopje, North Macedonia; 47 Secciones de Alergia Adultos y Pediátrica, Hospital Italiano de Buenos Aires, Buenos Aires, Argentina; 48 Department of Clinical and Environmental Allergology, Jagiellonian University Medical College, Krakow, Poland; 49 Division of Allergy and Clinical Immunology, Department of Medicine, University of Cape Town, Cape Town, South Africa; 50 Allergy and Immunology Unit, University of Cape Town Lung Institute, Cape Town, South Africa; 51 Department of Medicine, Ribeirão Preto Medical School, University of São Paulo, Ribeirão Preto, Brazil; 52 Faculty of Medicine, Pediatric Allergy and Asthma Division, Hacettepe University, Ankara, Turkey; 53 Asthma, Allergy and Immunology Service, Escola Superior de Ciências da Santa Casa de Misericórdia de Vitória, Espírito Santo, Brazil; 54 TUM School of Medicine and Health, Department of Otorhinolaryngology, Head and Neck Surgery, TUM University Hospital, Munich, Germany; 55 Department of Allergology, Medical University of Sofia, University Hospital Alexandrovska, Sofia, Bulgaria; 56 Division of Allergy and Clinical Immunology, Department of Medicine, Faculty of Medicine Siriraj Hospital, Mahidol University, Bangkok, Thailand; 57 Division of Rheumatology and Clinical Immunology, Department of Medicine, Queen Mary Hospital, Hong Kong, Hong Kong; 58 Istanbul Faculty of Medicine, Department of Pediatric Allergy and Immunology, Istanbul University, Istanbul, Turkey; 59 Cerrahpasa Faculty of Medicine, Department of Pediatric Allergy and Immunology, Istanbul University Cerrahpasa, Istanbul, Turkey; 60 CSL Behring, King of Prussia, PA USA; 61 CSL Behring LLC, Bern, Switzerland; 62 Division of Rheumatology and Clinical Immunology, Department of Medicine, The University of Hong Kong, Hong Kong, Hong Kong

**Keywords:** Hereditary angioedema, Guidelines, Management, Treatment goals, Unmet needs

## Abstract

**Background:**

Hereditary angioedema (HAE) is a rare and potentially life-threatening genetic disorder characterized by unpredictable attacks of angioedema. MENTALIST (UnMEt Needs in herediTAry angioedema—a gLobal physIcian perSpecTive) is the first international survey uncovering unmet needs and identifying barriers to optimal management in HAE following the latest update of the World Allergy Organization (WAO)/European Academy of Allergy and Clinical Immunology (EAACI) HAE guidelines.

**Methods:**

This web-based survey comprised 24 questions on HAE management and unmet needs. HAE-expert physicians from the Angioedema Centers of Reference and Excellence network ranked unmet needs according to their own perspectives and their patients’ perspectives, using a 10-point Likert scale ranging from 0 (not a challenge/unmet need at all) to 10 (huge challenge/unmet need).

**Results:**

Of 64 respondents from 32 countries, most (91%) had > 5 years of experience in managing HAE. Overall, 48% of respondents (*n* = 31/64) reported that < 50% of their patients had achieved the WAO/EAACI HAE treatment goals of total disease control and “normalization” of life at the time of the survey. Implementation of consensus recommendations was found to be inconsistent across regions. Gaps in non–HAE-expert physician knowledge, treatment costs, and reimbursement for long-term prophylaxis were the highest-priority challenges according to the respondents. Burden of disease remains a challenge among patients, as reported by their physicians.

**Conclusions:**

The MENTALIST findings highlight a need for removal of barriers to HAE treatment goals and propose a call to action to improve access to treatments, for greater provision of education for physicians and patients, critical collaboration with patient organizations and industry stakeholders and ultimately to optimize HAE care.

**Supplementary Information:**

The online version contains supplementary material available at 10.1186/s13023-025-03739-8.

## Introduction

Hereditary angioedema (HAE) is a rare, autosomal dominant, and potentially life-threatening disorder characterized by recurrent, unpredictable attacks of cutaneous or submucosal angioedema [1]. The most common HAE types (with an estimated global prevalence of approximately 1 in 50,000–100,000) are caused by C1 inhibitor (C1INH) deficiency, i.e., HAE-C1INH-Type1, or dysfunction, i.e., HAE-C1INH-Type2, leading to uncontrolled bradykinin production, vascular permeability, and subsequent angioedema [1–5]. Rarer and genetically heterogeneous HAE types are characterized by normal C1INH levels (HAE-nC1INH) [6, 7]. HAE-nC1INH pathophysiology is complex, encompassing multiple different genetic mutations causing abnormal proteins: while some subtypes are directly associated with bradykinin overproduction, others may be associated with reduced regulation of endothelial permeability or other mechanisms [6, 8].

Historically, patients with HAE have faced several unmet needs, including misdiagnosis (or delayed diagnosis), inadequate access to specialized care and limited access to treatment, frequent attacks, and impaired quality of life (QoL) [3, 9–12]. Although asphyxiation from laryngeal edema is rare, a review of historical real-world data estimated a rate of one death for every 20 patients, suggesting deaths may still occur [13]. Similarly, patients with HAE may undergo unnecessary invasive diagnostic and surgical procedures due to the confounding symptoms of abdominal attacks [14].

The unpredictability and severity of HAE attacks place significant physical and emotional burden on patients and their caregivers, whose activities of daily living and relationships are seriously impacted [15]. The frequency of HAE attacks increases anxiety and depression, reduces QoL, and is the main driver of poor disease control [11, 16–18].

In 2022, the World Allergy Organization (WAO)/European Academy of Allergy and Clinical Immunology (EAACI) published updated HAE treatment guidelines, providing recommendations for the management of HAE [1]. Long-term prophylaxis (LTP) treatment was indicated as a critical means of achieving the goals of total control of the disease (no HAE attacks) and “normalization” of life [1]. Emphasis was also placed on diagnosing HAE early and optimizing HAE management using validated patient-reported outcome measures (PROMs), such as the Angioedema Quality of Life Questionnaire, Angioedema Control Test, Angioedema Activity Score, and Hereditary Angioedema Quality of Life Questionnaire [1]. Another validated, psychometrically sound questionnaire for HAE-C1INH, the Hereditary Angioedema Activity Score, also provides a linear measure of disease activity [19].

Despite recent treatment advances, reports indicate that some patients do not achieve WAO/EAACI HAE guideline treatment goals and experience significant disease burden [18, 20]. Evidence from the Asia–Pacific region (including Australia, China, India, Japan, Malaysia, Mongolia, Philippines, Singapore, Taiwan, Thailand, South Korea, and Vietnam) recently highlighted gross disparities in access to testing within the region, resulting in underestimated prevalence compared with global rates (0.02 in 100,000) [21, 22]. Lack of diagnostic facilities and patient advocacy groups were also associated with delayed diagnosis and limited access to treatments [21]. Regional studies stressed substantial differences in country-specific needs, demographics, comorbidity incidences, and the impact of treatment decisions on healthcare resource utilization (HCRU) and societal costs, and demonstrate a remaining burden of disease despite improvements in HAE management [21, 23, 24].

These disparities contribute to large variation in disease burden and unmet needs, highlighting the need for global data [18, 21]. To provide an international perspective on the level of unmet need in the care of patients with HAE, HAE-expert physicians participated in the web-based MENTALIST (UnMEt Needs in herediTAry angioedema—a gLobal physIcian perSpecTive) survey. The purpose of this survey was to provide an updated overview of critical current unmet needs at an international level within ACARE, including barriers to achieving WAO/EAACI HAE treatment goals [1], 2 years after the publication of the WAO/EAACI HAE treatment guidelines. To our knowledge, this is the first survey capturing physicians’ and physician-reported patients’ feedback in a single manuscript for patients with HAE-C1INH as well as HAE-nC1INH.

## Methods

### Survey development and data collection

This web-based survey on unmet needs consisted of 24 multiple-choice, open-ended, and scale-based questions, and was developed and implemented by Angioedema Centers of Reference and Excellence (ACARE) in collaboration with CSL Behring. This project aligns with ACARE’s vision of increasing the knowledge of angioedema by means of research and education, and promoting excellence in angioedema management, as well as awareness of angioedema by advocacy activities [25]. The survey was hosted on REDCap®, a secure online application for surveys and databases [26]. HAE-expert physicians practicing in certified ACAREs (84 centers in 35 countries) or applicant ACAREs (22 centers, as of September 2023) were invited via email, newsletter, and social media channels to participate voluntarily in the survey from September 1 to 30, 2023. HAE-expert physicians ranked unmet needs and barriers to achieving treatment goals that were identified through a literature search (see the supporting information in the Supplementary Information). The ranking was based on a 10-point Likert scale ranging from 0 (not a challenge/unmet need at all) to 10 (huge challenge/unmet need) and was completed according to physicians’ own perspectives and their patients’ perspectives, the latter collected as feedback received at patient visits by means of open conversations between the patient and physician. This indirect approach to collection of patient feedback was sought to facilitate simultaneous collection of physicians’ and patients’ perspectives. Thus, patient feedback was not collected systematically with a set questionnaire.

### Data analysis

For each survey question, responses were anonymized and analyzed descriptively (mean with standard deviation or median with interquartile range [IQR], respectively) using R (R Foundation for Statistical Computing) version 4.2.3. Only descriptive statistical analyses were conducted. Although responses from unsubmitted surveys were excluded, completion of the survey was not mandatory for submission, and thus percentages were calculated according to the number of responses obtained and not the overall respondent population. Unmet needs were categorized according to proportions of respondent-level scores (low challenge: 0 to < 3.333; medium challenge: ≥ 3.333 to  < 6.667; high challenge:  ≥ 6.667 to 10). Unmet needs were also identified in the context of access to LTP therapies, as identified through the survey. Similar unmet needs were grouped by overarching categories (knowledge and education, disease burden, treatment, disease management, and patient-specific unmet needs). Percentages were rounded to the nearest whole number. Any instance of duplicated survey response was investigated with the physician via email prior to anonymization of the data and analysis (see the supporting information in the Supplementary Information).

## Results

### Characteristics of physician respondents

Of 84 physicians who initiated the questionnaire, 64 respondents from 32 countries submitted the survey and comprised the analysis population (Table 1; Supplementary Table S1). All 64 respondents provided answers on the general information section of the survey (e.g., practice characteristics, access to treatments and testing, use of PROMs). Respondents addressed questions on unmet needs as per their clinical experience in HAE-C1INH (*n* = 63) and HAE-nC1INH (*n* = 50): for this reason, the number of respondents is specified along with percentages throughout this section. One respondent was contacted via email upon submitting responses to the survey twice and, following their decision, the data from one of the submitted surveys were discarded. Reasons for unsubmitted survey responses were not investigated directly with the 20 physicians who did not submit their responses to the survey. Most respondents were from Europe (*n* = 29/64, 46%); South America and the Middle East were the second most represented regions (*n* = 11/64, 17% each); North America, East Asia, Africa, and Australia accounted for 20% of respondents (*n* = 13/64). Most respondents practiced in certified ACAREs (*n* = 49/64, 77%) and had > 5 years of experience in managing HAE (*n* = 58/64, 91%). At the time of the survey, respondents managed a median (IQR) of 40 patients (10–83) with HAE yearly, of whom 3 (1–6) per year were newly diagnosed. Overall, 47 of 64 respondents (73%) managed both pediatric and adult patients; 63 (98%) treated patients with HAE-C1INH and 50 (78%) also treated patients with HAE-nC1INH. Demographics and practice characteristics of respondents are summarized in Table 1.

**Table 1:** Demographic and characteristics of respondents

Characteristics	Physicians *N* = 64, *n* (%)
Regions*	
Western Europe (Austria, Denmark, France, Germany, Italy, Portugal, Spain, UK)	21 (33)
Eastern Europe (Bulgaria, Croatia, Czech Republic, Hungary, Macedonia, Poland, Russia)	8 (13)
South America (Argentina, Brazil, Peru)	11 (17)
Middle East (Kuwait, Oman, Qatar, Turkey, United Arab Emirates)	11 (17)
North America (Canada, USA)	4 (6)
East Asia (China, India, Japan, Thailand)	4 (6)
Africa (South Africa, Tunisia)	3 (5)
Australia	2 (3)
**Practice settings**	
ACARE	49 (77)
ACARE applicant	15 (23)
University clinic	36 (56)
Private practice	13 (20)
Public hospital	24 (38)
Private hospital	5 (8)
Others (i.e., National Research Center)	1 (2)
**Specialty**	
Allergy/immunology	52 (81)
Dermatology	13 (20)
Pediatrics	4 (6)
ENT	3 (5)
Rheumatology	2 (3)
Other (i.e., internal medicine)	4 (6)
**Years in practice**	
> 30 years	7 (11)
> 20 years	17 (27)
> 10 years	18 (28)
5–10 years	16 (25)
1–5 years	6 (9)
**Patient population**	
Adults only	14 (22)
Both pediatric and adult patients	47 (73)
Pediatric patients	3 (5)
**HAE type**	
HAE-C1INH	63 (98)
HAE-nC1INH	50 (78)
**Access to ODTs**	
Recombinant C1INH (IV)	22 (34)
Plasma-derived C1INH (IV)	57 (89)
Icatibant	55 (86)
**Access to LTP therapies**	
Lanadelumab (SC)	49 (77)
Plasma-derived C1INH (IV)	48 (75)
Plasma-derived C1INH (SC)	36 (56)
Berotralstat	32 (50)
Androgens	52 (81)
Tranexamic acid	58 (91)
Other LTP	4 (6)
**Access to testing**	
Complement C4	63 (98)
C1INH levels	58 (91)
C1INH functional levels	59 (92)
C1q	54 (84)
Genetic testing for HAE-nC1INH mutations	44 (69)
Whole genome sequencing	21 (32)
Other tests	4 (6)

*A list of respondents by country is available in Supplementary Table S1

Abbreviations: *ACARE* Angioedema Centers of Reference and Excellence, *C1q* complement component 1q, *C4* complement component 4, *C1INH* C1 inhibitor, *ENT* ear, nose, and throat, *HAE-C1INH* HAE due to deficiency or dysfunction of C1 inhibitor, *HAE-nC1INH* HAE due to normal C1INH, *IV* intravenous, *LTP* long-term prophylaxis, *ODT* on-demand treatment, *SC* subcutaneous

Data regarding access to testing and availability of HAE guidelines and educational programs for physicians and/or patients are summarized in Supplementary Table S2 and Supplementary Figs. S1 and S2.

### Availability of first-line on-demand treatments and first-line LTP therapies for HAE

Consistent with local market approvals, respondents indicated the availability of several on-demand treatments (ODTs) and LTP in their countries (Table 1). Intravenous plasma-derived C1INH was the most widely available first-line ODT. The availability of first-line LTP differed, with respondents indicating having access to the following therapies: lanadelumab (*n* = 49/64, 77%); plasma-derived C1INH intravenous (*n* = 48/64, 75%) or subcutaneous (*n* = 36/64, 56%); and berotralstat (*n* = 32/64, 50%).

### Use of PROMs

Most respondents had access to PROMs (Supplementary Table S3), with over 75% utilizing them in their practice. Only 38% of respondents (*n* = 24/64) used PROMs at every patient visit, and 39% (*n* = 25/64) used them often. Additionally, 19% (*n* = 12/64) indicated that they used PROMs rarely, while 5% (*n* = 3/64) had never used them in their clinical practice at the time of the survey. The proportion of respondents using PROMs in certified ACAREs was marginally higher than in applicant ACAREs (78% vs 73%) (Supplementary Fig. S3).

### Current unmet needs in HAE-C1INH and HAE-nC1INH

**Fig. 1:**
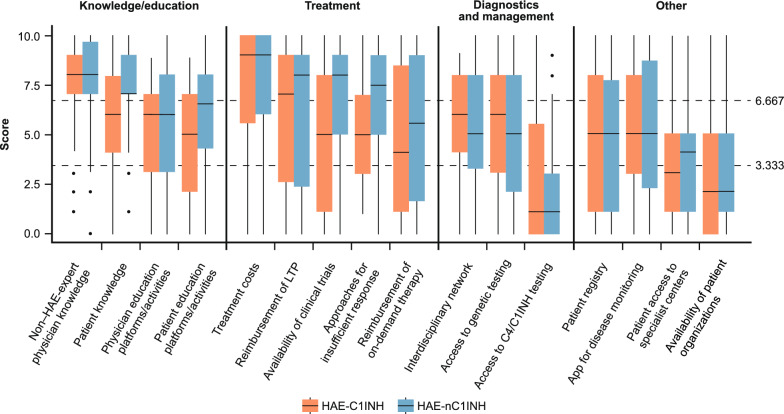
Physician perspectives: Ranking of challenges and unmet needs physicians face in treating patients with HAE-C1INH and HAE-nC1INH (overall population). Median values are represented by a solid line in the center of the box. Boxes indicate the IQR with whiskers extending to 1.5 × IQR. Outlier responses are reported as scatter points. The two scatter horizontal lines at 3.333 and 6.667 separate the three unmet need categories (Low: <3.333; medium: ≥3.333 to <6.667; high: ≥6.667). Abbreviations: *C4* complement component 4, *C1INH* C1 inhibitor, *HAE* hereditary angioedema, *HAE-C1INH* HAE due to deficiency or dysfunction of C1 inhibitor, *HAE-nC1INH* HAE due to normal C1INH, *IQR* interquartile range, *LTP* long-term prophylaxis

**Fig. 2:**
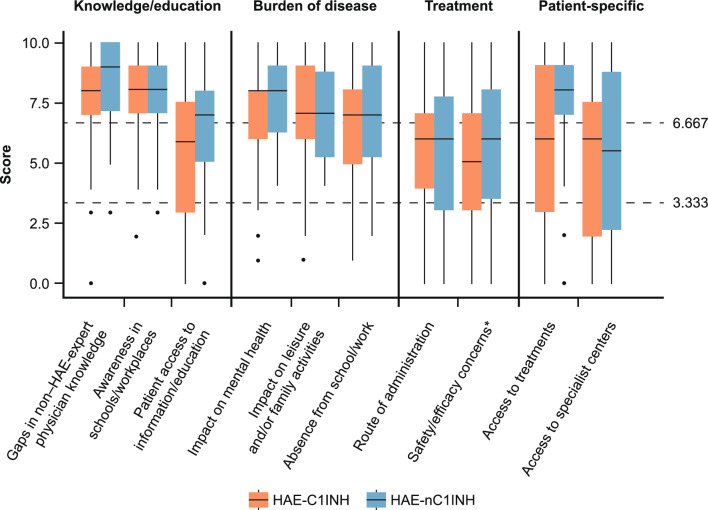
Physician-reported patient perspectives: Ranking of challenges and unmet needs patients with HAE-C1INH and HAE-nC1INH report to their treating physicians (overall population). Median values are represented by a solid line in the center of the box. Boxes indicate the IQR with whiskers extending to 1.5 × IQR. Outlier responses are reported as scatter points. The two scatter horizontal lines at 3.333 and 6.667 separate the three unmet need categories (Low: <3.333; medium: ≥3.333 to <6.667; high: ≥6.667). *Including concerns about effectiveness of currently available treatments. Abbreviations: *HAE* hereditary angioedema, *HAE-C1INH* HAE due to deficiency or dysfunction of C1 inhibitor, *HAE-nC1INH* HAE due to normal C1 inhibitor, *IQR* interquartile range

Of the 64 respondents, 63 addressed questions regarding HAE-C1INH, while 50 addressed questions regarding HAE-nC1INH. Overall, high unmet needs were comparable for HAE-C1INH and HAE-nC1INH, with only subtle differences. Unmet needs by category are shown in Figs. 1 and 2, and corresponding scores for each unmet need are shown in Supplementary Tables S4 and S5 (survey questions are provided in the Supplementary Information).

#### Physician perspectives: “Knowledge/education” and “Treatment” categories included the highest unmet needs

This section reports the unmet needs ranked by respondents according to the physicians’ perspectives (Fig. 1; Supplementary Table S4). Most respondents agreed that gaps in knowledge about HAE and its treatment among non–HAE-expert physicians constituted a high unmet need both in HAE-C1INH (*n* = 48/63, 76%) and HAE-nC1INH (*n* = 42/50, 84%). Additionally, most respondents (*n* = 39/50, 78%) perceived gaps in patient knowledge as a high unmet need in HAE-nC1INH; however, less than half of the respondents (*n* = 29/63, 46%) shared the same view regarding HAE-C1INH. Consistent with this finding, the need for patient education platforms or activities was reported to be slightly higher for patients with HAE-nC1INH than HAE-C1INH, with median (IQR) scores of 6.5 (4.0–8.0) and 5.0 (2.5–7.0), respectively.

The “Treatment” category accounted for most high-priority unmet needs among respondents, with treatment costs being the highest scoring unmet need in both HAE-C1INH (*n* = 46/63, 73%) and HAE-nC1INH (n = 36/50, 72%). Reimbursement of LTP scored highly in both HAE-C1INH (*n* = 32/63, 51%) and HAE-nC1INH (*n* = 31/50, 62%), whereas availability of clinical trials and approaches for insufficient responses recorded higher proportions of high unmet need responses for HAE-nC1INH (*n* = 32/50, 64% and *n* = 31/50, 62%, respectively) than for HAE-C1INH (*n* = 24/64, 38% each) (Fig. 1; Supplementary Table S4). Access to specialist centers, availability of patient organizations, and access to complement component 4 (C4)/C1INH testing were ranked as low-to-moderate unmet needs.

Through the survey, six respondents (9%) from Brazil (*n* = 1/64), South Africa (*n* = 1/64), Peru (*n* = 2/64), and Tunisia (*n* = 2/64) reported having access to second-line LTP only (tranexamic acid, 100% [6/6]; androgens, 83% [5/6]; other LTP, 17% [1/6]). These respondents reported a substantial number of high unmet needs in most categories. Median scores of 10 (representing the highest challenge) for both HAE-C1INH and HAE-nC1INH were reported for treatment costs, reimbursement of LTP, and reimbursement of ODTs. In contrast with medium-to-low median scores recorded in the overall population of respondents (Fig. 1; Supplementary Table S4), unmet needs regarding diagnostics and management (e.g. access to C4/C1INH or genetic testing), and access to apps for disease monitoring and to specialist centers scored highly (medians ranging between 7 and 10) among respondents with sole access to second-line LTP.

#### Patient perspectives as reported by their physicians: “Education,” “Burden of disease,” and “Patient-specific” categories were the highest unmet needs

This section reports unmet needs ranked according to patient perspectives as reported by their physicians (Fig. 2; Supplementary Table S5). In the “Burden of disease” category, the impact of HAE on patient mental health was perceived as a substantial challenge for all patients with HAE (Supplementary Table S5). Gaps in non–HAE-expert physician knowledge was ranked as the highest unmet need in HAE-nC1INH (*n* = 45, 90%) according to physician-reported patient perspectives (Fig. 2; Supplementary Table S5). Awareness of HAE in schools and workplaces received high unmet need scores both in HAE-C1INH (*n* = 48/63, 76%) and HAE-nC1INH (*n* = 38/50, 76%). Patient access to information and access to treatments were perceived as a higher priority in HAE-nC1INH than in HAE-C1INH (Supplementary Table S5).

Consistent with the observations for unmet needs from the physician perspective, respondents with sole access to second-line treatments reported that patients with HAE-nC1INH perceived a higher burden of disease than the overall respondent population, with medians in that category ranging between 9 and 10. Moreover, while treatment safety and efficacy concerns were generally reported as a medium unmet need by the overall respondent population (Fig. 2), respondents with sole access to second-line treatments indicated these as high unmet needs for both HAE-C1INH and HAE-nC1INH according to their patients’ perspectives (medians ranging between 8.5 and 10).

### Barriers to achieving the WAO/EAACI HAE treatment goals

**Fig. 3:**
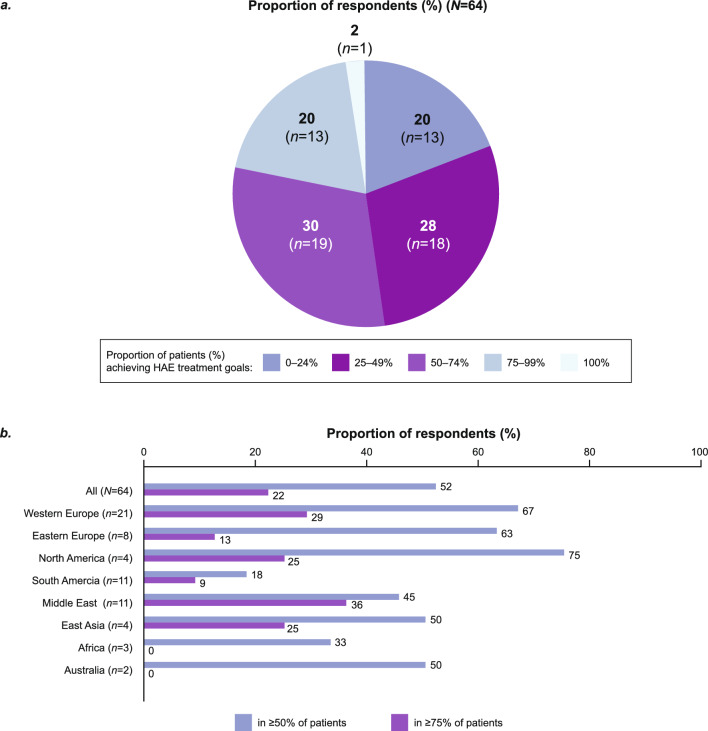
Proportion of respondents who reported achieving HAE treatment goals of total control of the disease and “normalization” of life in their patients: data by **a** overall population and **b** region. Abbreviation: *HAE* hereditary angioedema

Overall, 48% of respondents (*n* = 31/64) reported that less than half of their patients (< 50%) had achieved the WAO/EAACI HAE treatment goals of total control of the disease and “normalization” of life at the time of the survey (Fig. 3). Most respondents in North America (*n* = 3/4, 75%), Western Europe (*n* = 14/21, 67%), and Eastern Europe (*n* = 5/8, 63%) estimated that ≥ 50% of their patients had achieved the HAE treatment goals at the time of the survey. Africa (*n* = 1/3, 33%) and South America (*n* = 2/11, 18%) had the lowest proportion of respondents achieving the treatment goals for ≥ 50% of their patients (Fig. 3). Notably, in the two African countries surveyed (Tunisia and South Africa), access to treatment was limited to only second-line LTP therapies.

**Fig. 4:**
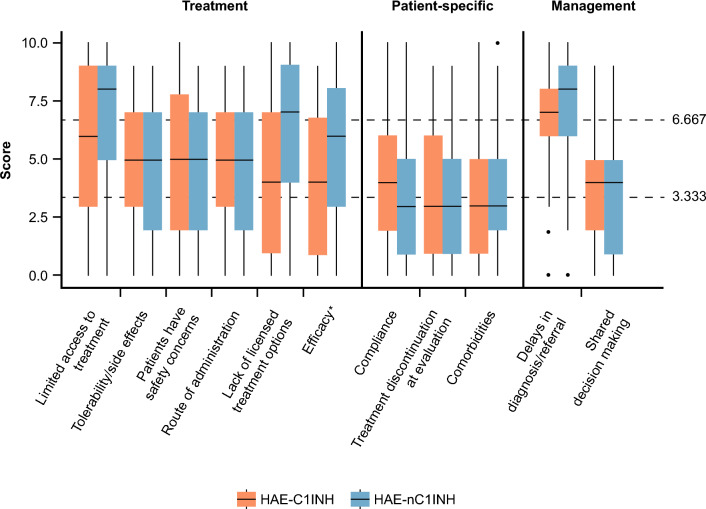
Ranking of barriers to achieving WAO/EAACI HAE treatment goals in patients with HAE-C1INH and HAE-nC1INH (overall population). Median values are represented by a solid line in the center of the box. Boxes indicate the IQR with whiskers extending to 1.5 × IQR. Outlier responses are reported as scatter points. The two scatter horizontal lines at 3.333 and 6.667 separate the three unmet need categories (Low: <3.333; medium: ≥3.333 to <6.667; high: ≥6.667). *Including concerns about effectiveness of currently available treatments. Abbreviations: *EAACI* European Academy of Allergy and Clinical Immunology, *HAE* hereditary angioedema, *HAE-C1INH* HAE due to deficiency or dysfunction of C1 inhibitor, *HAE-nC1INH* HAE due to normal C1 inhibitor, *IQR* interquartile range, *WAO* World Allergy Organization

Barriers and challenges to achieving HAE treatment goals by overarching category and by score are reported in Fig. 4 and Supplementary Table S6, respectively.

Delays in diagnosis and/or referral scored the highest among the barriers to achieving HAE treatment goals in patients with HAE-C1INH (*n* = 40/62, 65%) and HAE-nC1INH (*n* = 34/49, 69%). While limited access to treatment options was experienced by most respondents (*n* = 31/49, 63%) as the second highest reason for not achieving HAE treatment goals in HAE-nC1INH, a lower proportion of respondents (*n* = 28/62, 45%) viewed this as a challenge in HAE-C1INH.

Although a lack of licensed treatment options was indicated as a substantial contributor to failing to achieve HAE treatment goals in HAE-nC1INH by over half of respondents (*n* = 25/49, 51%), only 31% of respondents (*n* = 19/62) believed this to be a critical factor in HAE-C1INH.

In countries with access solely to second-line LTP (*n* = 6), a lack of licensed treatment options for HAE-C1INH scored as one of the biggest challenges (median: 10).

## Discussion

The MENTALIST survey highlights high-priority unmet needs from the perspectives of ACARE HAE-expert physicians and their patients and provides insights on how many patients achieve HAE treatment goals, 2 years after the latest update of the WAO/EAACI HAE treatment guidelines [1].

Gaps in non–HAE-expert physician knowledge and patient access to education were identified as highest-priority unmet needs and represent a barrier to optimal care. While non–HAE-expert physician knowledge on HAE could not be directly assessed with this survey, ACARE HAE-expert physicians could provide an important assessment of the degree of disease awareness in primary care based on their experience with patient referrals from non-HAE-expert physicians. Thus, the opinions provided by HAE-expert physicians in the MENTALIST survey support published reports of low disease awareness in primary care leading to missed symptoms, misdiagnosis, delayed diagnosis, and subsequent suboptimal treatment [22]. Diagnostic delays can lead to unnecessary surgical procedures [14], untreated life-threatening attacks, and even patient mortality [27]. Therefore, educational programs targeted to non–HAE-expert physicians are needed to decrease delays in diagnosis and/or referrals, which also emerged from this survey as the principal barriers to achieving the WAO/EAACI HAE treatment goals. Furthermore, educational programs should be extended to the general population and include specific initiatives for patients, their families, and caregivers, to enhance their understanding of HAE symptoms, the associated mortality risk, and the importance of prompt urgent medical care. To improve the current situation, close cooperation between HAE-expert physician networks (e.g., ACARE), patient organizations (e.g., HAE International, The US Hereditary Angioedema Association), and industry stakeholders is imperative to create online educational platforms, campaigns in schools, or face-to-face initiatives to raise awareness of HAE.

Alongside education, treatment costs and reimbursement for LTP were also highest-priority unmet needs according to HAE-expert physicians, indicating that more needs to be done to remove barriers to access to fully reimbursed treatments. Limited access to treatment was also perceived as a critical barrier to achieving the WAO/EAACI HAE treatment goals. As emphasized in the WAO/EAACI HAE guidelines, LTP should be encouraged as the best means by which to achieve total disease control [1]. Although LTP may be perceived as more expensive than ODT, regional cost-effectiveness studies in LTP users have already demonstrated lower HCRU and ODT costs over time [24], and have also shown that well-controlled HAE leads to higher productivity and lower medical and care costs than poorly controlled HAE [18, 21]. As HAE management is acutely expensive, we encourage physicians to assess barriers to treatment access or reimbursement for LTP with HCRU analyses that would account for economic resources at a regional or country level. Notably, this type of analyses was not performed in this study due to the intrinsic imbalance in the geographical representation of ACARE physician respondents; for this reason, a careful approach should be applied when designing any studies to ensure a balanced geographical representation among physician respondents. Therefore, we recommend the collection and publication of additional HCRU evidence and concerted action between physicians, industry, and patient organizations, which may encourage governments to extend reimbursements to new LTPs. While diagnostics and management were identified as medium-to-low unmet needs by the physician respondents, we acknowledge the need for homogeneous access to testing and closer inter-center collaboration across the ACARE network.

Results show that patients with HAE still experience high burden of disease, with attacks having a detrimental effect on mental health; disease awareness in schools and workplaces and absenteeism are perceived as major challenges. This evidence aligns with reports of limitations in patients’ activities of daily living, care needs (e.g., dental or surgical procedures), and career choices [4, 28]. A recent narrative review extensively described the psychological burden of HAE, and reported higher levels of anxiety and depression in patients with HAE compared with the general population; this review also outlined other disorders such as mania, anger, sleep disorders, somatic symptoms, and impaired personality functioning [12].

The lack of clinical studies and approved therapies for HAE-nC1INH is a long-standing concern, which was again emphasized in this study [6]. The solution to this concern seems remote; however, as more patients are identified with de novo genetic causes of HAE-nC1INH, the organization of clinical trials with stringent inclusion criteria may lead to the identification of efficacious therapies and subsequent approvals by regulatory agencies. Notably, the creation of global patient registries like the Chronic Angioedema Registry (CARE) [29] by the ACARE Network may facilitate recruitment into these clinical trials and better monitoring of disease and treatment outcomes.

Data from this survey showed that a substantial proportion of patients does not achieve the WAO/EAACI HAE treatment goals worldwide. Although the survey indicated similar degrees of total disease control achieved in Europe or North America as well as Asia, the number of physician respondents representing the two regions was not comparable. Therefore, additional surveys with comparable numbers of physician respondents are needed to provide a more accurate inter-regional evaluation of the extent of disease control achieved in patients with HAE.

Despite international consensus on the approach to management of HAE, this survey shows inconsistent implementation of the consensus recommendations across regions. While acknowledging the intrinsic limitations posed by restricted access to reimbursed LTP (even in high-resource countries, for example in the USA in the form of prior authorizations or in countries like Australia where a substantial number of HAE attacks must be experienced by patients before access to reimbursed LTP can be granted), this survey shows that there is an opportunity to implement concrete actions aimed at improving assessment of disease control. Namely, PROMs are widely available [30], but less than half of respondents make use of them at every patient visit. This is important, as monitoring of disease activity leads to objective evaluations of disease control, allows dynamic treatment optimization, and aids evidence collection to support insurance coverage or marketing authorization applications [31]. In the digital era, patient disease monitoring apps may represent a practical solution, especially among young patients, and are now successfully integrated into the care pathway for chronic spontaneous urticaria [32]. In addition, specialized healthcare providers, such as HAE-trained nurses, could strengthen the partnership with patients, facilitate regular monitoring of PROMs, and maximize patient outcomes through shared decision-making. Official documentation of angioedema episodes should always be encouraged (whether through self-reporting in a diary, specialized applications, and emergency or out-patient clinic records). These records are also crucial for reimbursement of HAE medications.

Observations from this survey could help HAE-expert physicians make informed recommendations for future updates to the WAO/EAACI HAE guidelines. As this survey was not powered to examine country-specific challenges in detail but to provide an overview of HAE management in ACARE, we encourage careful evaluation of regional challenges via targeted research and surveys, and of the applicability of treatment recommendations as well as the potential barriers to their implementation. We also recommend early engagement with target stakeholders (e.g., patient organizations, local healthcare networks) for effective adoption of international guidance, and tailoring of recommendations to diverse settings, considering the variations in treatment access and costs that exist between countries and among individual patients.

To the best of our knowledge, the MENTALIST survey represents the first international physician survey to provide an overview of the unmet needs in HAE following the latest update of the WAO/EAACI HAE treatment guidelines. An additional strength is the identification of both physician and physician-reported patient perspectives within the same survey, which were possible to collect only via the pivotal role and international reach of the ACARE network. Furthermore, insights into unmet needs specific to HAE-nC1INH, an often under-researched patient group in the past, were also identified. In terms of international representation, the Middle East and South American experiences were well represented, with each region comprising 17% of respondents.

The study has some limitations. Firstly, patient-specific unmet needs were not measured with a set questionnaire and indirectly reported via the physician rather than by patients themselves. In future studies, this limitation could be mitigated by closely collaborating with international (e.g. HAE International/US Hereditary Angioedema Association) or national patient organizations to expand survey reach and enable accurate representation through the collection of direct patient feedback via standardized tools [33, 34]. As for other surveys, there is a limitation regarding potential for recall and selection biases, as respondents were more likely to remember details they themselves considered to be important. Secondly, an imbalance in the global distribution of respondents makes the data potentially over-representative of the ACARE experience in Europe, and regional unmet needs being potentially generalized or understated. Lastly, these results mostly reflect the views and experience of HAE-expert physicians practicing in accredited ACAREs (or in applicant ACAREs), where HAE clinical experience is well established. Therefore, non–HAE-expert physicians in other settings may face even greater or different unmet needs from those described in this analysis.

## Conclusions and call to action

The MENTALIST survey showed that a substantial proportion of patients with HAE do not currently achieve the HAE treatment goals of total disease control and “normalization” of life specified in the latest update of the WAO/EAACI HAE treatment guidelines. Delayed diagnosis and limited access to treatments were identified as critical barriers to HAE treatment goals. Gaps in non–HAE-expert physician and patient knowledge, treatment costs, and reimbursements for LTP were identified as highest-priority unmet needs. To address these critical needs, we urge close cooperation between HAE-expert physicians, industry stakeholders, and patient organizations to bridge gaps in physician and patient education. Physicians are also encouraged to use PROMs frequently, not only to optimize care but also to collect standardized evidence that can be leveraged in policy-making to increase access to new treatment strategies.

## Supplementary Information

Additional file1 (DOCX 228 kb)

## Data Availability

Data-sharing requests will be reviewed case by case by CSL Behring.

## Abbreviations

ACARE: Angioedema Centers of Reference and Excellence
C1INH: C1 inhibitor
C1q: Complement component 1q
C4: Complement component 4
EAACI: European Academy of Allergy and Clinical Immunology
ENT: Ear, nose, and throat
HAE: Hereditary angioedema
HAE-C1INH-Type1: HAE due to C1INH deficiency Type 1
HAE-C1INH-Type2: HAE due to C1INH deficiency Type 2
HAE-nC1INH: HAE due to normal C1 inhibitor
HCRU: Healthcare resource utilization
IQR: Interquartile range
IV: Intravenous
LTP: Long-term prophylaxis
MENTALIST: UnMEt Needs in herediTAry angioedema—a gLobal physIcian perspective
ODT: On-demand treatment
PROM: Patient-reported outcome measure
QoL: Quality of life
SC: Subcutaneous
WAO: World Allergy Organization
