# Supplementary material for: Unmet needs in hereditary angioedema: an international survey of physicians
Source: Orphanet J Rare Dis. 2025 Jul 28;20:383. doi: 10.1186/s13023-025-03739-8 (PMC12306030; doi:10.1186/s13023-025-03739-8)
Supplement: Supplementary file 1 — Additional file1 (DOCX 228 kb) [file 13023_2025_3739_MOESM1_ESM.docx]

# Supplementary information

**Title:** Unmet needs in hereditary angioedema: An international survey of physicians

Buttgereit et al.

## Supporting information

**Literature search on unmet needs**

To formulate the MENTALIST (UnMEt Needs in herediTAry angioedema – a gLobal physIcian perSpecTive) survey, common unmet needs were identified via a review of recent literature. A total of 25 hits related to unmet needs were obtained via a PubMed search (January 2020–June 2023). Five additional congress abstracts and one publication on unmet needs in HAE were identified as potentially suitable via desk research. Of the total, 10 were excluded to remove any duplicates and articles with limited discussion on unmet needs.


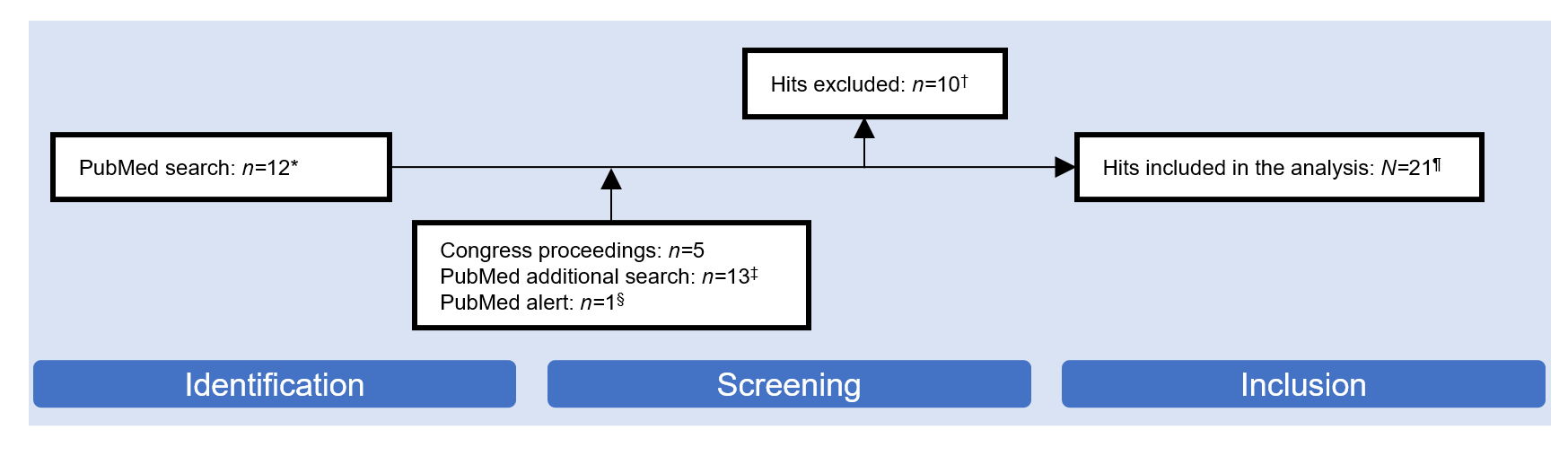


*Search string: ‘unmet need*[Title/Abstract] AND hereditary angioedema[Title/Abstract]’; ^†^Unmet needs only marginally discussed (*n*=8) and duplicates (*n*=2); ^‡^Search string: ‘challenge*[Title/Abstract] AND hereditary angioedema[Title/Abstract]’; ^§^Alert from search: ‘hereditary angioedema’.
Abbreviation: *HAE* hereditary angioedema.

**Duplicated survey response**

One instance of survey duplication occurred (65 completed questionnaires from 64 respondents). The matter was investigated via email, and the physician clarified a potential error with the first submission; for this reason, responses from the first submission were discarded from the analysis.

### Supplementary Table 1 Geographic location of physician respondents

| **Country** | ***N*=64**  ***n* (%)** |
| --- | --- |
| Argentina | 2 (3) |
| Australia | 2 (3) |
| Austria | 2 (3) |
| Brazil | 7 (11) |
| Bulgaria | 1 (2) |
| Canada | 2 (3) |
| China | 2 (3) |
| Croatia | 2 (3) |
| Czech Republic | 1 (2) |
| Denmark | 1 (2) |
| France | 4 (6) |
| Germany | 8 (13) |
| Hungary | 1 (2) |
| India | 1 (2) |
| Italy | 2 (3) |
| Japan | 1 (2) |
| Kuwait | 1 (2) |
| Macedonia | 1 (2) |
| Oman | 2 (3) |
| Peru | 1 (2) |
| Poland | 2 (3) |
| Portugal | 1 (2) |
| Qatar | 3 (5) |
| Russia | 2 (3) |
| South Africa | 1 (2) |
| Spain | 2 (3) |
| Thailand | 1 (2) |
| Tunisia | 2 (3) |
| Turkey | 2 (3) |
| United Arab Emirates | 1 (2) |
| United Kingdom | 1 (2) |
| United States | 2 (3) |

### Supplementary Table 2 Physicians with access to testing for HAE-nC1INH mutations

| **Mutations** | ***n* (%)** |
| --- | --- |
| *FXII* | 42 (66) |
| *PLG* | 33 (52) |
| *ANGPT* | 28 (44) |
| *KNG* | 26 (41) |
| *MYOF* | 21 (33) |
| *HSST* | 16 (25) |

Abbreviations: *ANGPT* angiopoietin-1 gene, *FXII* factor XII gene, *HAE-nC1INH* hereditary angioedema with normal levels of C1 inhibitor, *HSST* heparan sulfate-glucosamine 3-*O*-sulfotransferase 6 gene, *KNG* kininogen-1 gene, *MYOF* myoferlin gene, *PLG* plasminogen gene.

### Supplementary Table 3 PROMs available to physician respondents

| **PROMs** | ***n* (%)** |
| --- | --- |
| AECT | 61 (95) |
| AAS | 55 (86) |
| AE-QoL | 53 (83) |
| HAE-QoL | 42 (66) |

Abbreviations: *AAS* Angioedema Activity Score, *AECT* Angioedema Control Test, *AE-QoL* Angioedema Quality of Life Questionnaire, *HAE-QoL* Hereditary Angioedema Quality of Life Questionnaire, *PROM* patient-reported outcome measure.

### Supplementary Table 4 Rankings of unmet needs from the physician perspective

|  | HAE-C1INH ***n*=63*** | | | | HAE-nC1INH ***n*=50*** | | | | |
| --- | --- | --- | --- | --- | --- | --- | --- | --- | --- |
| Unmet need | Median  (IQR) score | Respondents per category, *n* (%) | | | Median  (IQR) score | Respondents per category, *n* (%) | | |  |
|  |  | High unmet need | Moderate unmet need | Low unmet need |  | High unmet need | Moderate unmet need | Low unmet need |  |
| Gaps in non–HAE-expert physician knowledge about HAE and its treatment | 8.0 (7.0–9.0) | 48 (76) | 11 (17) | 4 (6) | 8.0 (7.0–10.0) | 42 (84) | 4 (8) | 4 (8) |  |
| Gaps in patient knowledge about HAE and its treatment | 6.0 (4.0–8.0) | 29 (46) | 20 (32) | 14 (22) | 7.0 (7.0–9.0) | 39 (78) | 8 (16) | 3 (6) |  |
| Physician education platforms/activities | 5.5 (3.0–7.0) | 20 (32) | 26 (41) | 17 (27) | 6.0 (3.0–8.0) | 24 (48) | 12 (24) | 14 (28) |  |
| Patient education platforms/activities | 5.0 (2.5–7.0) | 17 (27) | 24 (38) | 22 (35) | 6.5 (4.0–8.0) | 25 (50) | 13 (26) | 12 (24) |  |
| Treatment costs | 9.0 (6.5–10.0) | 46 (73) | 9 (14) | 8 (13) | 9.0 (6.0–10.0) | 36 (72) | 8 (16) | 6 (12) |  |
| Reimbursement  of LTP | 7.0 (3.0–9.5) | 32 (51) | 10 (16) | 21 (33) | 8.0 (2.0–9.0) | 31 (62) | 4 (8) | 15 (30) |  |
| Availability of clinical trials | 5.0 (2.0–8.0) | 24 (38) | 11 (17) | 28 (44) | 8.0 (5.0–9.0) | 32 (64) | 9 (18) | 9 (18) |  |
| Approaches for insufficient response | 5.0 (3.5–8.0) | 24 (38) | 22 (35) | 17 (27) | 7.5 (5.0–9.0) | 31 (62) | 11 (22) | 8 (16) |  |
| Reimbursement of on-demand treatment | 5.0 (1.0–9.0) | 26 (41) | 6 (10) | 31 (49) | 5.5 (1.0–9.0) | 24 (48) | 7 (14) | 19 (38) |  |
| Interdisciplinary network | 6.0 (4.0–8.0) | 28 (44) | 21 (33) | 14 (22) | 5.0 (3.0–8.0) | 22 (44) | 15 (30) | 13 (26) |  |
| Access to genetic testing | 6.0 (3.0–8.0) | 29 (46) | 12 (19) | 22 (35) | 5.0 (2.0–8.0) | 22 (44) | 9 (18) | 19 (38) |  |
| Access to C1INH/C4 testing | 1.0 (0.0–5.5) | 10 (16) | 11 (17) | 42 (67) | 1.0 (0–3.0) | 5 (10) | 7 (14) | 38 (76) |  |
| Patient registry | 5.0 (1.0–8.0) | 20 (32) | 14 (22) | 29 (46) | 5.0 (1.0–8.0) | 19 (38) | 11 (22) | 20 (40) |  |
| App for disease monitoring | 5.0 (3.0–8.0) | 28 (44) | 15 (24) | 20 (32) | 5.0 (2.0–9.0) | 22 (44) | 14 (28) | 14 (28) |  |
| Patient access to specialist centers | 3.0 (1.0–5.0) | 12 (20) | 18 (29) | 33 (52) | 4.0 (1.0–5.0) | 7 (14) | 19 (38) | 24 (48) |  |
| Patient organization | 2.0 (0–5.0) | 5 (8) | 12 (19) | 46 (73) | 2.0 (1.0–5.0) | 8 (16) | 12 (24) | 30 (60) |  |

*Percentages based on non-missing responses.

Abbreviations: *C1INH* C1 inhibitor, *C4* complement component 4, *HAE* hereditary angioedema, *HAE-C1INH* HAE with deficiency or dysfunction of C1 inhibitor, *HAE-nC1INH* HAE with normal levels of C1 inhibitor, *IQR* interquartile range, *LTP* long-term prophylaxis.

### Supplementary Table 5 Rankings of unmet needs from the patient perspective, as reported by their physicians

|  | HAE-C1INH ***n*=63*** | | | | HAE-nC1INH ***n*=50*** | | | |
| --- | --- | --- | --- | --- | --- | --- | --- | --- |
| Unmet need | Median (IQR) score | Respondents per category, *n* (%) | | | Median (IQR) score | Respondents per category, *n* (%) | | |
|  |  | High unmet need | Moderate unmet need | Low unmet need |  | High unmet need | Moderate unmet need | Low unmet need |
| Gaps in non–HAE-expert physician knowledge about HAE and its treatment | 8.0 (7.0–9.0) | 51 (81) | 8 (13) | 4 (6) | 9.0 (7.0–10.0) | 45 (90) | 4 (8) | 1 (2) |
| Awareness of HAE in schools/workplaces | 8.0 (7.0–9.0) | 48 (76) | 14 (22) | 1 (2) | 8.0 (7.0–9.0) | 38 (76) | 11 (22) | 1 (2) |
| Patient access to information/education | 6.0 (3.0–7.5) | 25 (40) | 20 (32) | 18 (29) | 7.0 (5.0–8.0) | 33 (66) | 11 (22) | 6 (12) |
| Impact on mental health | 8.0 (6.0–8.0) | 42 (67) | 15 (24) | 6 (10) | 8.0 (6.0–9.0) | 37 (74) | 13 (26) | 0 |
| Impact on leisure and/or family activities | 7.0 (6.0–9.0) | 43 (68) | 14 (22) | 6 (10) | 7.0 (5.0–9.0) | 32 (64) | 18 (36) | 0 |
| Absence from school/work | 7.0 (5.0–8.0) | 42 (67) | 12 (19) | 9 (14) | 7.0 (5.0–9.0) | 30 (60) | 17 (34) | 3 (6) |
| Route of administration | 6.0 (4.0–7.0) | 26 (41) | 23 (37) | 14 (22) | 6.0 (3.0–8.0) | 20 (40) | 13 (26) | 17 (34) |
| Safety/efficacy concerns | 5.0 (3.0–7.0) | 21 (33) | 20 (32) | 22 (35) | 6.0 (3.0–8.0) | 23 (46) | 14 (28) | 13 (26) |
| Access to treatments | 6.5 (3.0–9.0) | 31 (49) | 14 (22) | 18 (29) | 8.0 (7.0–9.0) | 38 (76) | 7 (14) | 5 (10) |
| Access to specialist centers | 5.5 (2.0–7.5) | 26 (41) | 17 (27) | 20 (32) | 5.5 (2.0–9.0) | 23 (46) | 10 (20) | 17 (34) |

*Percentages based on non-missing responses.

Abbreviations: *HAE* hereditary angioedema, *HAE-C1INH* HAE with deficiency or dysfunction of C1 inhibitor, *HAE-nC1INH* HAE with normal levels of C1 inhibitor, *IQR* interquartile range.

### Supplementary Table 6 Barriers of challenges to achieving WAO/EAACI HAE treatment goals

|  | HAE-C1INH ***n*=62*** | | | | HAE-nC1INH ***n*=49*** | | | | |
| --- | --- | --- | --- | --- | --- | --- | --- | --- | --- |
| Reason or challenge | Median  (IQR) score | Respondents per category, *n* (%) | | | Median (IQR) score | Respondents per category, *n* (%) | | | |
|  |  | High/substantial challenge | Moderate challenge | Low challenge |  | High/substantial challenge | Moderate challenge | Low challenge |  |
| Limited access to treatment | 6.0 (3.0–9.0) | 28 (45) | 15 (24) | 19 (31) | 8.0 (5.0–9.0) | 31 (63) | 7 (14) | 11 (22) |  |
| Tolerability/side effects | 5.0 (3.0–7.0) | 17 (27) | 23 (37) | 22 (35) | 5.0 (2.0–7.0) | 15 (31) | 15 (31) | 19 (39) |  |
| Patients have safety concerns | 5.0 (2.0–8.0) | 24 (39) | 12 (19) | 26 (42) | 5.0 (2.0–7.0) | 17 (35) | 12 (24) | 20 (41) |  |
| Route of administration | 5.0 (3.0–7.0) | 23 (37) | 19 (31) | 20 (32) | 5.0 (2.0–7.0) | 14 (29) | 15 (31) | 20 (41) |  |
| Lack of licensed treatment options | 4.0 (1.0–7.0) | 19 (31) | 13 (21) | 30 (48) | 7.0 (4.0–9.0) | 25 (51) | 12 (24) | 12 (24) |  |
| Efficacy | 4.0 (1.0–7.0) | 16 (26) | 19 (31) | 27 (44) | 6.0 (3.0–8.0) | 19 (39) | 15 (31) | 15 (31) |  |
| Compliance | 4.0 (2.0–6.0) | 15 (24) | 18 (29) | 29 (47) | 3.0 (1.0–5.0) | 8 (16) | 11 (22) | 30 (61) |  |
| Treatment discontinuation at evaluation | 3.0 (1.0–6.0) | 12 (19) | 17 (27) | 33 (53) | 3.0 (1.0–5.0) | 7 (14) | 15 (31) | 27 (55) |  |
| Comorbidities | 3.0 (1.0–5.0) | 9 (15) | 14 (23) | 39 (63) | 3.0 (2.0–5.0) | 9 (18) | 14 (29) | 26 (53) |  |
| Delays in diagnosis/ referral | 7.0 (6.0–8.0) | 40 (65) | 17 (27) | 5 (8) | 8.0 (6.0–9.0) | 34 (69) | 10 (20) | 5 (10) |  |
| Shared decision making | 4.0 (2.0–5.0) | 9 (15) | 23 (37) | 30 (48) | 4.0 (1.0–5.0) | 7 (14) | 19 (39) | 23 (47) |  |

*Percentages based on non-missing responses.

Abbreviations: *EAACI* European Academy of Allergy and Clinical Immunology, *HAE* hereditary angioedema, *HAE-C1INH* HAE with deficiency or dysfunction of C1 inhibitor, *HAE-nC1INH* HAE with normal levels of C1 inhibitor, *IQR* interquartile range, *WAO* World Allergy Organization.

## Supplementary figure legends

### Supplementary Fig. 1 Proportion of physicians with access to biochemical and genetic testing by geographical region.

Abbreviations: *ANGPT* angiopoietin-1 gene, *FXII* factor XII gene, *C1INH* C1 inhibitor, *C4* complement component 4, *HSST* heparan sulfate-glucosamine 3-*O*-sulfotransferase 6 gene, *KNG* kininogen-1 gene, *MYOF* myoferlin gene, *PLG* plasminogen gene.

### Supplementary Fig. 2 Proportion of physicians indicating availability of regional/national HAE guidelines, education programs for physicians and patients, national patient organizations, patient-monitoring apps, and patient registries by geographical region.

Abbreviation: *HAE* hereditary angioedema.

### Supplementary Fig. 3 Proportion of physicians indicating the frequency of use of PROMs by center type and geographical region.

Abbreviations: *ACARE* Angioedema Centers of Reference and Excellence, *PROM* patient-reported outcome measure.

## MENTALIST survey

Dear Colleague,

Please answer the following 24 questions, which will take about 15–20 minutes.

First you will find general questions on the management of hereditary angioedema (HAE), regardless of disease subtype and the age of patients you see in your clinics. Further questions focus specifically on unmet needs in HAE in your country. We know your time is valuable, and we highly appreciate your help to improve the lives of patients with HAE.

If you wish, your contribution will be acknowledged in the publication of the results of this survey.

**General information**

1. What is your country of residence? (Please choose only one, from the following list)

(Please choose from the following list with countries from A–Z)

1. In which city do you practice as a physician?

(Please write down)

1. Optional: Please write down your first name and surname if you want your contribution to be acknowledged in the publication

(First name + surname)

1. What is your work environment?

(Please choose all that apply)

- ACARE (Angioedema Center of Reference and Excellence)
- University clinic
- Private practice
- Private hospital
- Public hospital
- Other (please specify): ____________________

1. What is your specialty?

(Please choose all that apply)

- Allergy/immunology
- Dermatology
- Hematology
- Oncology
- ENT
- Pediatrics
- Rheumatology
- Neurology
- General practice
- Gastroenterology
- Other (please specify): ________________

1. How many years of experience do you have in treating HAE patients?

(Single choice)

- <1 year
- 1–5 years
- 5–10 years
- >10 years
- >20 years
- >30 years

1. What is the estimated total number of HAE patients (including HAE-C1INH-Type 1/2 and HAE with normal C1INH) you see per year?

XX patients

1. Please estimate how many new HAE patients (confirmed diagnosis of HAE including HAE-C1INH-Type 1/2 and/or HAE with normal C1INH) do you see per year?

- 0
- 1
- 2–5
- 6–10
- >10
- >20
- >30

1. Do other specialists refer patients with suspected HAE to you?

(Please choose all that apply)

- No
- Yes, allergists/clinical immunologists
- Yes, dermatologists
- Yes, hematologists
- Yes, oncologists
- Yes, rheumatologists
- Yes, ENTs
- Yes, neurologists
- Yes, general practitioners
- Yes, gastroenterologists
- Yes, pediatricians
- Yes, endocrinologists
- Yes, other (please specify): ________________________________

1. Do you refer HAE patients to other specialists?
   (Please choose all that apply)

- No *(If no, skip next question)*
- Yes, allergists/clinical immunologists
- Yes, dermatologists
- Yes, hematologists
- Yes, oncologists
- Yes, rheumatologists
- Yes, ENTs
- Yes neurologists
- Yes, general practitioners
- Yes, gastroenterologists
- Yes, pediatricians
- Yes, orthopedics
- Yes, endocrinologists
- Yes, other (please specify): _______________________________

1. If you refer HAE patients to other specialists, what are the reasons?
   (Please choose all that apply)

- Diagnostic work-up
- Second opinion
- Treatment initiation
- Treatment optimization
- It is a specialized angioedema center, e.g., ACARE
- Other (please specify): ___________________________

1. What population of HAE patients do you see?

(Single choice)

- Children only
- Adults only
- Both, children and adults

1. What types of HAE do your patients have?

- HAE-C1INH-Type 1/2 x%
- HAE with normal C1INH x%

1. What subtypes of HAE with normal C1INH values do your patients have? (Please choose all that apply) *(This question can only be answered if you have provided a percentage for patients who have HAE with normal C1INH values in question 13)*

- HAE-FXII
- HAE-PLG
- HAE-ANGPT
- HAE-KNG
- HAE-MYOF
- HAE-HSST
- HAE-UNK

1. What diagnostic tests for HAE are accessible in your center?

(Please choose all that apply)

- Complement C4
- C1INH levels
- C1INH function
- C1q
- Genetic testing for HAE-C1INH mutations
- Genetic testing for HAE-ANGPT mutations
- Genetic testing for HAE-FXII mutations
- Genetic testing for HAE-PLG mutations
- Genetic testing for HAE-KNG mutations
- Genetic testing for HAE-MYOF mutations
- Genetic testing for HAE-HSST mutations
- Whole genome sequencing
- Other (please specify): ___________________________

1. What therapeutic options for on-demand treatment of acute HAE attacks are available in your country?

(Please choose all that apply)

- Plasma-derived C1INH (Cinryze^®^, Berinert^®^), IV
- Recombinant C1INH (Ruconest^®^), IV
- Icatibant (i.e., Firazyr^®^), SC
- Ecallantide (Kalbitor^®^), SC
- Fresh frozen plasma, IV
- Solvent-detergent plasma, IV
- Other (please specify): ____________________

1. What therapeutic options for long-term prophylaxis for HAE are available in your country?

(Please choose all that apply)

- Plasma-derived C1INH (Cinryze^®^, Berinert^®^), IV

- Plasma-derived C1INH (Berinert^®^, Haegarda^®^), SC

- Lanadelumab (Takhzyro^®^), SC

- Berotralstat (Orladeyo^®^), oral

- Androgens, oral

- Tranexamic acid, oral

- Other (please specify): ____________________________

1. What is available in your country?

- National/regional HAE guideline yes/no/don’t know

- Patient registry yes/no/don’t know

- Patient app for disease monitoring yes/no/don’t know

- Physician education programs for HAE yes/no/don’t know

- Patient education programs for HAE yes/no/don’t know

- National patient organization for HAE yes/no/don’t know

1. A. What patient reported outcome measures (PROMs) for the use in HAE are available in your country?

- AE-QoL (Angioedema Quality of Life Questionnaire) yes/no/don’t know
- AECT (Angioedema Control Test) yes/no/don’t know
- AAS (Angioedema Activity Score) yes/no/don’t know
- HAE-QoL (HAE Quality of Life Questionnaire) yes/no/don’t know
- Other (please specify): ____________________________

19. B. How often do you use PROMs in clinical practice for HAE patients?

- Not at all

- Rarely

- Often

- At every visit

**Challenges and unmet needs for HAE-treating physicians**

1. A. From the physician perspective, what challenges/unmet needs do you see in the management of **HAE-C1INH-Type 1/2 patients** in your country?

*(Please rank the following items from 0 = not a challenge/unmet need at all to 10 = huge challenge/unmet need)*

- Gaps in patient knowledge about HAE and its treatment 0–10
- Gaps in non–HAE-expert physician knowledge about HAE and its treatment 0–10
- Multidisciplinary network is missing or not active 0–10
- Lack of patient education platforms and activities 0–10
- Lack of physician education platforms and activities 0–10
- Patient organization missing or not active 0–10
- Angioedema specialist centers are difficult to access for patients 0–10
- Access to C1INH/C4 testing 0–10
- Access to testing for HAE mutations 0–10
- Challenges in the reimbursement of on-demand treatment options 0–10
- Challenges in the reimbursement of long-term prophylaxis treatment options 0–10
- Approaches to patients with insufficient response to current treatment options 0–10
- High treatment costs 0–10
- App for disease monitoring not available 0–10
- Not enough clinical trials for HAE 0–10
- Patient registry missing or not accessible 0–10
- Other: ____________________________ 0–10
- Other: ____________________________ 0–10
- Other: ____________________________ 0–10

B. From the physician perspective, what challenges/unmet needs do you see in the management of your **HAE patients with normal C1INH** in your country?

*(Please rank the following items from 0 = not a challenge/unmet need at all to 10 = huge challenge/unmet need)*

- Gaps in patient knowledge about HAE and its treatment 0–10
- Gaps in non–HAE-expert physician knowledge about HAE and its treatment 0–10
- Multidisciplinary network is missing or not active 0–10
- Lack of patient education platforms and activities 0–10
- Lack of physician education platforms and activities 0–10
- Patient organization missing or not active 0–10
- Angioedema specialist centers are difficult to access for patients 0–10
- Access to C1INH/C4 testing 0–10
- Access to testing for HAE mutations 0–10
- Challenges in the reimbursement of on-demand treatment options 0–10
- Challenges in the reimbursement of long-term prophylaxis treatment options 0–10
- Approaches to patients with insufficient response to current treatment options 0–10
- High treatment costs 0–10
- App for disease monitoring not available 0–10
- Not enough clinical trials for HAE 0–10
- Patient registry missing or not accessible 0–10
- Other: ____________________________ 0–10
- Other: ____________________________ 0–10
- Other: ____________________________ 0–10

**Challenges and unmet needs HAE patients report to their physician**

1. What challenges/unmet needs do **HAE-C1INH-Type 1/2 patients report** living with the disease in your country?

*(Please rank the following items from 0 = not a challenge/unmet need at all to 10 = huge challenge/unmet need)*

- Knowledge gaps of non–HAE-expert physicians in HAE and its treatment 0–10
- Angioedema specialist centers challenging to access 0–10
- Absence from school/work due to angioedema attacks 0–10
- Impact on leisure and/or family activities 0–10
- Impact on mental health 0–10
- Challenges in accessing information and education on HAE and treatments 0–10
- Challenges in accessing treatments 0–10
- Safety and/or efficacy concerns about treatment 0–10
- Challenges with route of administration of treatment 0–10
- Lack of HAE awareness in schools/workplaces 0–10
- Other: ______________________ 0–10
- Other: ______________________ 0–10
- Other: ______________________ 0–10

1. What challenges/unmet needs do **HAE patients with normal C1INH report** living with the disease in your country?

*(Please rank the following items from 0 = not a challenge/unmet need at all to 10 = huge challenge/unmet need)*

- Knowledge gaps of non–HAE-expert physicians in HAE and its treatment 0–10
- Angioedema specialist centers challenging to access 0–10
- Absence from school/work due to angioedema attacks 0–10
- Impact on leisure and/or family activities 0–10
- Impact on mental health 0–10
- Challenges in accessing information and education on HAE and treatments 0–10
- Challenges in accessing treatments 0–10
- Safety and/or efficacy concerns about treatment 0–10
- Challenges with route of administration of treatment 0–10
- Lack of HAE awareness in schools/workplaces 0–10
- Other: ______________________ 0–10
- Other: ______________________ 0–10
- Other: ______________________ 0–10

23. Please estimate how many of your **HAE patients (including HAE-C1INH and HAE with normal C1INH)** in your country do meet the treatment goals according to the current international WAO/EAACI guideline for the management of HAE?

- 0%–25%

- 25%–50%

- 50%–75%

- 75%–100%

- 100%

If the answer to Q23 is not 100%, Q23A and 23B will follow

23A. What may be the reasons why **HAE-C1INH-Type 1/2 patients** do not meet the treatment goals according to the international WAO/EAACI guideline in your country?

*Please rank the following items from 0 = not relevant at all to 10 = maximum relevant*

- Patients have safety concerns about available treatment options 0–10
- Patients experience tolerability issues or side effects 0–10
- Patients have comorbidities that do not allow optimal treatment 0–10
- Patients face challenges in the administration of the treatment options available 0–10
- Treatment options available are not effective 0–10
- Lack of licensed treatment options 0–10
- Issues with access to treatment options 0–10
- Challenges in shared decision making between patient and physician 0–10
- Patient discontinued treatment at the time of evaluation 0–10
- Patient shows low compliance 0–10
- Delays in diagnosis and/or referral 0–10
- Other (free text + ranking) 0–10

23B. What may be the reasons why **HAE patients with normal C1INH** do not meet the treatment goals according to the international WAO/EAACI guideline in your country?

*Please rank the following items from 0 = not relevant at all to 10 = maximum relevant*

- Patients have safety concerns about available treatment options 0–10
- Patients experience tolerability issues or side effects 0–10
- Patients have comorbidities that do not allow optimal treatment 0–10
- Patients face challenges in the administration of the treatment options available 0–10
- Treatment options available are not effective 0–10
- Lack of licensed treatment options 0–10
- Issues with access to treatment options 0–10
- Challenges in shared decision making between patient and physician 0–10
- Patient discontinued treatment at the time of evaluation 0–10
- Patient shows low compliance 0–10
- Delays in diagnosis and/or referral 0–10
- Other (free text + ranking) 0–10

24. Lastly, is there anything else that has not been addressed by research in HAE and you would like explored in the future?

(Free text answer)

**Thank you for participating in our survey!**

**Your answers are very important to identify the worldwide challenges and unmet needs in HAE. Thank you for helping with improving the medical care of patients.**
